# Continuous culture of urine-derived bladder cancer cells for precision medicine

**DOI:** 10.1007/s13238-019-0649-5

**Published:** 2019-07-25

**Authors:** Shuai Jiang, Jiaqi Wang, Chen Yang, Renke Tan, Jun Hou, Yuan Shi, Huihui Zhang, Shiyu Ma, Jianan Wang, Mengmeng Zhang, George Philips, Zengxia Li, Jian Ma, Wanjun Yu, Guohua Wang, Yuanming Wu, Richard Schlegel, Huina Wang, Shanbo Cao, Jianming Guo, Xuefeng Liu, Yongjun Dang

**Affiliations:** 1 Department of Urology, Zhongshan Hospital, Fudan University, Shanghai, 200032 China; 2 Key Laboratory of Metabolism and Molecular Medicine, Ministry of Education and Department of Biochemistry and Molecular Biology, School of Basic Medical Sciences, Fudan University, Shanghai, 200032 China; 3 Department of Pathology, Zhongshan Hospital, Fudan University, Shanghai, 200032 China; 4 Department of Respiratory and Critical Care Medicine, Yinzhou Hospital Affiliated to Medical school of Ningbo University, Ningbo, 315000 China; 5 School of Computer Science and Technology, Harbin Institute of Technology, Harbin, 150000 China; 6 AcornMed Biotechnology Co., Ltd, Beijing, 100176 China; 7 Department of Oncology, Lombardi Comprehensive Cancer Center, Georgetown University Medical School, MedStar Georgetown University Hospital, Washington, DC 20001 USA; 8 Department of Biochemistry and Molecular Biology and Center for DNA Typing, Fourth Military Medical University, Xi’an, 710000 China; 9 Department of Pathology, Center for Cell Reprogramming, Lombardi Comprehensive Cancer Center, Georgetown University Medical School, Washington, DC 20001 USA

**Dear Editor,**

Bladder cancer is the most common type of genitourinary cancer in China, with an estimated 80,500 new cases and 32,900 related deaths in 2015 alone (Chen et al., 2016). Unlike many other cancers, there has been no significant improvement in survival rates for bladder cancer over the last three decades. Specific treatment regimens for bladder cancer and their efficacy vary depending not only on clinical stages, but also on associated risk factors and other personal clinical characteristics. Patients with non-muscle invasive bladder cancer (NMIBC) have a high 5-year recurrence rate of 60%–70% (Berdik, 2017) and those with muscle invasive bladder cancer (MIBC) has a relatively poor prognosis with approximately 65% risk of death within 5-year follow-up (Kamat et al., 2016). Therefore, there is an urgent need to develop models for bladder cancer to screen for rational treatment strategies by personalized medicine to improve the clinical assessment and treatment of bladder cancer.

Conditional reprogramming (CR) technique is a transformational method of cell culture that allows rapid and efficient generation of cells from normal and tumor tissues (Jin et al., 2018). Patient-derived cells can grow indefinitely without genetic manipulation (Liu et al., 2012) and has been proven useful in basic and clinical research in many kinds of cancers (Saeed et al., 2017). In previous cases, CR cultures (CRCs) were generated using biopsy or surgery samples collected through invasive approaches. While it might not be easily performed or accepted in some cases. Liquid biopsies have been proven to provide equally accurate and dynamic clinical information and can capture the complex genetic mutations of profiles of primary and metastatic tumors (Karachaliou et al., 2015). Urine is the most convenient source of liquid biopsies and has been extensively explored for clinical diagnosis of bladder cancer by cytology and biomarkers (Di Meo et al., 2017). Therefore, the establishment of primary urine-derived cancer cell models using CR technique might be ideal in clinical practice.

We adapted a CR technique to explore the possibility of establishing bladder cancer cells from patients’ tumor tissues and urine samples and applied the cultures for whole exome sequencing (WES) and drug testing (Fig. 1A). All patients were diagnosed as bladder cancer by pathology, detailed information of patients is summarized in Fig. 1B and Table S1. The overall success rate of culturing urine CRCs was 83.3% (50/60), specifically, high grade bladder cancer was 85.4% (41/48) and low grade bladder cancer was 75.0% (9/12) (Fig. 1C). Moreover, we did not observe the bias of success rates of culturing urine CRCs with respect to gender, pathology group, disease status or age from all bladder cancer patients (Fig. S1). We would like to expand our sample size in the future, especially the tumor in situ bladder cancer. For patients with systemic metastasis of recurrent tumors, we are also trying to culture circulating tumor cells.

**Figure 1:**
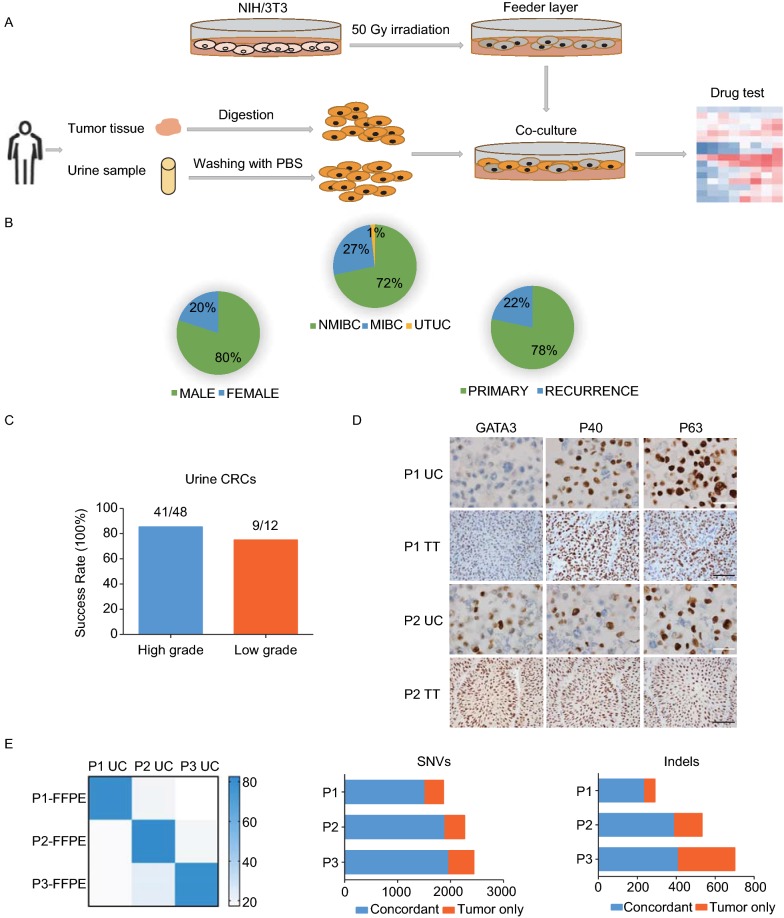
**Establishment and characterization of cell cultures from urine and tumor samples of bladder cancer.** (A) Workflow of the CR method for collection of urine and tissue samples and establishment of primary bladder cancer cell cultures. Bladder cancer tissue samples were obtained from surgery or cystoscopy biopsy and the clean-catch, midstream urine samples were collected before tumor resection or before surgery into 50-mL sterile tubes after clean the urethral area of the patient. The CRCs were generated from the urine and tissue samples using cocultured with irradiated NIH/3T3 feeder cells and ROCK inhibitor (Y-27632) and the drug sensitivity were measured on clinical oncology drugs. (B) Pie charts show the classification of all urine-provided patients based on gender (left), pathology group (middle), and disease status (right). The patients enrolled include 80% (48/60) of male and 20% (12/60) of female. Non-muscle invasive bladder cancer (NMIBC), muscle invasive bladder cancer (MIBC), and upper tract urothelial carcinoma (UTUC) accounted for 72% (43/60), 27% (16/60), and 1% (1/60), respectively, among which 78% (47/60) were primary and 22% (13/60) were recurrence. (C) Cells from high grade (85.4%, 41/48) showed relatively high success rate than low grade (75.0%, 9/12) bladder cancer. Tumor grades are arranged in columns, success rates are arranged in rows. (D) Immunohistochemistry staining of urine CRCs from patient 1 and 2 and the parental tissues with the indicated markers (GATA3, P40. P63) (representative image of *n* = 3 independent experiments). The scale bars indicate 50 μm (CRCs) and 200 μm (tissues). UC = urine CRC; TT = tumor tissue; P = patient; (E) Correlation heat map between the variants identified in urine CRCs and formalin-fixed paraffin-embedded (FFPE) tissue specimens by whole-exome sequencing analysis and concordance of SNVs and indels detected in the parental tumor tissues and corresponding urine CRCs. Number of mutations are arranged in columns, patients are arranged in rows. Tumor only or concordant mutations are displayed by different colors in the bottom panel

Primary cells isolated from urine and tumor samples both rapidly formed CRC colonies and representative compact spheroids in 3D culture (Fig. S2A). HE staining results showed that the CRCs exhibited obvious tumor cell properties as with their corresponding tumor pathological section (Fig. S2B). To identify the molecular characteristics, we performed IHC staining to determine the expression of three cell type-specific markers (GATA3, P40, and P63) in urine CRCs. The expression of all three markers were high in the urine CRC of patient 2 and two markers were high (P40, P63) in patient 1, which were consistent with their parental tumor tissues (Fig. 1D). Furthermore, Transwell assays in urine CRC showed different migration abilities for each sample. (Fig. S3).

We also carried out WES analysis in urine CRCs and compared the results to the corresponding parental tumors. In total, CR cells shared 79.7%–82.6% of their variation profile with primary tumor tissues (Fig. 1E). Similarly, the analysis of the mutation ratio for both patients’ tissues and corresponding urine CRCs confirmed that both single nucleotide variants (SNVs) and insertions and deletions (indels) in the original tissues were well retained during culturing (Fig. 1E). Parts of the landscape of common somatic mutations among paired urine CRCs, tumor CRCs, and tumor tissues for the same patient are shown in Fig. S5. Some common mutations were also found in these three patient-derived tumor tissues, as shown in Fig. S6. We also identified some SNVs that are not represented in the primary tumor. This may be due to the failure to propagate some of the clones from the primary tumor or because these SNVs in reality may be present in the primary tumors, but at a very low level under the detection limit and were able to selectively grow in the CR method. Recently it was shown that CR technology was successful in identifying low frequency high impact actionable mutations in primary breast cancer and liver metastasis patients (Anjanappa et al., 2018). Thus, these models can be useful not only for drug discovery, but also to simulate the drug resistance and to better understand the inter-play between various clonal populations within a tumor that leads to tumor progression and metastasis. STR analysis confirmed CRCs can retain their origin and genetic stability during prolonged passaging and rule out the possibility of contamination by other cell lines (Tables S2 and S3). There were also several minor differences, which are usually caused by tumor genomic microsatellite instability and heterogeneity, but this discrepancy does not affect the identity of the origin. Furthermore, we detected the mutation of telomerase reverse transcriptase (TERT) promoter in CRCs and the parental tumor tissue (Fig. S4 and Table S4) and the results are consistent with previous studies reporting that C124T is a more prevalent mutation as compared to C146T, with a 62.6% mutation rate in bladder cancer (946/1,511) (Vinagre et al., 2013).

We validated that urine CRCs maintain molecular characteristics and genetic alterations of original tissues and thus they will be an ideal model for determining the drug sensitivities. Each CRC was treated with 64 clinical oncology drugs for 72 h before cell viability was measured. Drug sensitivities were represented by IC_50_ and DSS (Table S5). Overall, CRCs were resistant to more than half of the drugs, with an IC_50_ greater than the maximum screening concentration. DSS of CRCs were used to draw the heatmap, except for all CRC-resistant drugs. The responses to these drugs revealed striking similarities and differences between different urine CRCs (Fig. 2A). Some CRCs showed high sensitivities to specific drugs, for example, urine CRC of patient 9 was highly sensitive to afatinib and lapatinib, which have the same target EGFR. Paclitaxel, docetaxel, and vinblastine sulfate are all microtubule inhibitors, we observed that urine CRCs of patient 5 and patient 8 both displayed significant responses to these three drugs. Notably, all urine CRCs showed high sensitivity to bortezomib, which is consistent with the previous reports that revealed bortezomib has significant antiproliferative activity in aggressive bladder cancer cells (Kamat et al., 2004). Coincidentally, bortezomib is in a clinical trial for patients with urothelial cancer, further suggesting that it may be a potentially effective drugs for bladder cancer. The DSS also showed relative correlations in urine and tumor CRCs from the same patients while distinguishing responses to certain drugs (Fig. 2A). A possible cause of these results is tumor heterogeneity.

**Figure 2:**
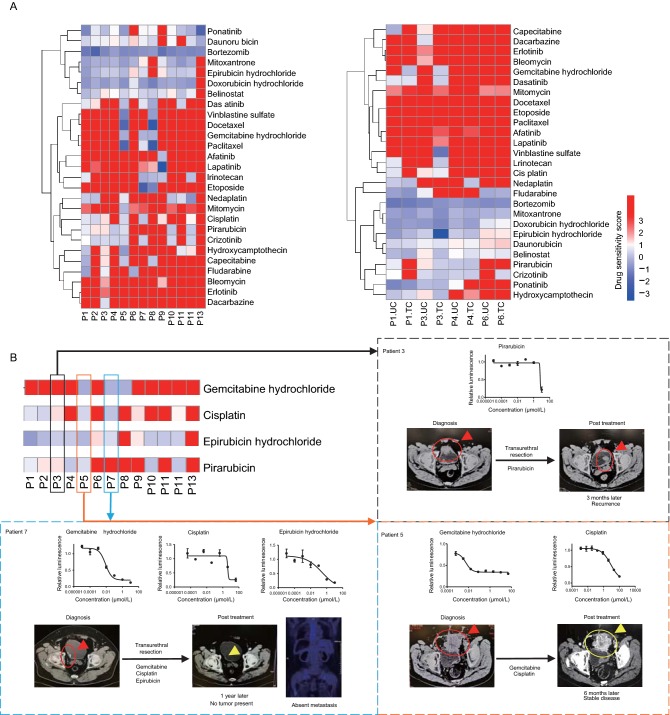
**Correlation of drug responses of CRCs and clinical outcomes of corresponding drug-treated patients.** (A) The heatmap of drug sensitivity scores (DSS) in urine CRCs derived from patient 1–13 and in urine and corresponding tumor CRCs of patient 1, 3, 4 and 6. DSS means log_10_ transformed fold-change of IC_50_ in comparison to 5637 cell line. Red represents higher DSS and blue represents lower DSS. The DSS was clustered by heatmap in R package (*n* = 3 independent experiments, the data indicates the mean). TC = tumor CRC. (B) The top left panel shows parts of heatmap of drug sensitivities of urine CRCs. The solid borders highlight patient 3, 5, and 7. Within the dotted borders, the patients’ dose-response curves for the indicated drugs are exhibited in the upper row, and the CT and PET-CT of the patients before and after treatment are shown in the lower row. The type of surgical treatment is shown on the black arrow and the drugs used are under the black arrow. Red circles and arrows indicate primary or recurrence tumors. Yellow circles and arrows represent response

Precision medicine based on genotype-informed therapies only have a median response rate of 54% in patients (Marquart et al., 2018). Also, tumors continue to evolve during cancer progression which leads to extensive tumor heterogeneity (Rubio-Perez et al., 2015). In our study, we can obtain urine samples at any time before and after treatment, which provides the possibility to obtain real-time pathological conditions. To translate our *ex vivo* analysis into a clinical framework, we compared CRC-based drug sensitivities with clinical responses in retrospective clinical studies. Here, we have demonstrated the clinical consistency in drug testing by urine CRCs (Figs. 2B and S7). Urine CRCs of patient 3 showed relatively low sensitivity to pirarubicin, which was consistent with clinical follow-up: patient 3’s tumor soon relapsed 3 months after transurethral resection of tumor(TURBT) followed by intravesical instillation of pirarubicin(40mg). Patient 5, who had been diagnosed with MIBC, showed relatively sensitivity to gemcitabine and cisplatin. In the absence of surgery, his CT obtained 6 months later showed stable disease (SD) after treatment with gemcitabine (1,000 mg/m^2^/intravenous) and cisplatin (70 mg/m^2^/intravenous). Another instance was demonstrated in patient 7, who showed obvious responses to gemcitabine (1,000 mg/m^2^/intravenous), cisplatin (70 mg/m^2^/intravenous), and epirubicin (40 mg/intravesical) treatments after transurethral resection. The CT and PET-CT obtained from a regular follow-up 1 year later exhibited no tumor present and absent metastasis, highlighting the accuracy of our drug screening results in relevance to clinical response. Patient 4 and 11 both accepted treatment with epirubicin after surgery and had no recurrence for 18 and 12 months, respectively, which was consistent with the high sensitivity of their corresponding urine CRCs to epirubicin. For patient 13, who was diagnosed with recurrent disease and treated with transurethral resection of bladder tumor (TUBRT) and epirubicin, relapsed soon 3 months later, and the urine CRC also displayed greatest drug resistance, not only to gemcitabine, but also to a wide range of drugs. Besides, we tested the sensitivity of patient 21’s urine CRC to gemcitabine and cisplatin alone, the drug resistance results were consistent with the clinical response of relapsing soon (Fig. S7). We also observed the opposite effects of drug sensitivity and clinical response on patient 1, who relapsed 6 months later after treatment with epirubicin. Our group is continuing expanding our sample size for further confirming and supporting of this application in clinical practice.

In conclusion, we successfully built up a novel, convenient model of urine CRCs that faithfully retains the molecular characteristics and genetic landscapes of the original tumor. The high success rate and rapid proliferation of urine CRCs implied they are suitable for large-scale drug testing. Certainly, correlating histological, genetic, and/or gene expression data to urine CRCs drug responses will further advance our molecular and functional understanding of bladder cancer. Also, the ability to form 3D spheroids indicating we can also combine the advantages of 3D tumor microenvironment simulation in future research. With a short timescale from establishment to drug testing, this novel *in vitro* bladder cancer system thus opens up new avenues for predicting patient-specific drug responses and creating personalized medicine into a reality.

## Electronic supplementary material

Below is the link to the electronic supplementary material.

Supplementary material 1 (PDF 1573 kb)
