# Supplementary material for: Continuous culture of urine-derived bladder cancer cells for precision medicine
Source: Protein Cell. 2019 Jul 25;10(12):902–7. doi: 10.1007/s13238-019-0649-5 (PMC6881267; doi:10.1007/s13238-019-0649-5)
Supplement: Supplementary file 1 — Supplementary material 1 (PDF 1573 kb) [file 13238_2019_649_MOESM1_ESM.pdf]

Materials and methods

Patients

All participating patients were diagnosed with bladder cancer, among which were 48 high grade and 12 low grade. Seven healthy volunteers were chosen as a control group. Urine and tumor samples were collected before clinical chemo-therapeutic treatment. Our study has been approved by Zhongshan Hospital Ethnics Committee (project number: B2016-148 and B2017-129R). All experimental protocols were carried out in accordance with the guidelines approved by the Zhongshan Hospital Ethics Committee. Informed consent was obtained from all of the patients in this study.

Urine and tissue processing

Urine samples of each patient were collected from clean, mid-stream urine before treatment. 50 ml of urine was collected in sterile tubes with penicillin/streptomycin mix (final concentration: 200U/mL penicillin and 200μg/mL streptomycin) and centrifuged at 1000 rpm for 10 min. The supernatant was aspirated, and the pellet was washed with PBS for three times before added into culture plates.

Bladder tumor tissues were collected from patients undergoing transurethral resection or cystoscopy and then transferred into a 15 ml tube with sterilized PBS. All samples were packaged on ice and the following steps were conducted in sterile conditions within 6 h. Tissues were minced with scalpels into small pieces, and parts of the tissues were saved for genomic DNA extraction and others were digested with

digestion buffer composed of dispase (Gibco), collagenase (Sigma) and hyaluronidase (Sigma). After dissociation, the cells were centrifuged at 1000 rpm for 5 min and the supernatant was discarded. Cell pellets were re-suspended in medium and transferred into plates for culturing.

Establishment of urine and tumor CRCs

For preparation of complete F medium, we mixed 373 ml of DMEM (Gibco) and 125 ml of F12 nutrient (Gibco) and added the following additions with final concentrations of 5 μg/ml insulin (Sigma-Aldrich), 0.125 ng/ml EGF (Sangon) and 25 ng/ml hydrocortisone (Sigma-Aldrich). The solution was filtered with a 0.2-μm sterile filter and stored at 4℃ for up to 2 weeks after adding the ROCK inhibitor Y-27632 (DC Chemicals) at a final concentration of 10 μM. NIH-3T3 cells were irradiated at a dose of 50 Gy as feeder cells. Cell pellets derived from urine and tumor samples were transferred into a 6 cm dish with feeder cells at a confluence of 1×10^4 cells/cm^2 in complete F medium. Formation of visible CRC colonies was monitored after 24-72 hours seeding. CRCs were differentially trypsinized to separate from feeder cells and passaged when reached 80% to 90% confluence using a 1:3 dilution and were given fresh medium every 2 to 3 days.

3D cell culture

The established CRCs were differentially trypsinized to be separated from feeder cells and counted using Cell Counter (Invitrogen). They were subsequently seeded onto round-bottomed non-tissue culture treated 96-well plates (Thermo) at a concentration

of 2500 cells/well in 100μL DMEM-F12 (Gibco), supplemented with 20% methyl cellulose stock solution and 10% FBS (Gibco). Spheroids were grown under standard culture conditions (5% CO2, at 37℃) and formed 3D cultures within 24 hours.

HE staining and immunohistochemistry

CRCs and tissues were fixed with 10% neutral buffered formalin, embedded in paraffin, and cut into 4 μm sections. The sections were stained with hematoxylin and eosin (H&E) following standard protocols. For immunohistochemistry, the sections were de-paraffinized, rehydrated and antigens were retrieved by heating the slides in 0.01 M sodium citrate (pH 6.0). The following antibodies were used: GATA3 (Gene Tech), P40 (DAKO), and P63 (MXB). Antibodies were visualized using 3,3'-diaminobenzidine (DAB) chromogen, counterstained with hematoxylin and mounted with DPX (Sigma-Aldrich).

Transwell assays

$2x10^4$ CRCs were seeded on the top chamber of an 8 μm 24 mm polycarbonate Transwell ® insert (Costar) in serum-free complete F medium while the bottom chamber contained complete F 20% FBS following an adapted protocol. The cells were allowed to migrate for 48 hours. The cells attached to the top of the membrane were removed using aseptic cotton carriers and rinsed with PBS. Lastly, the bottom of the membrane was fixed with 95% alcohol, stained with 0.1% crystal violet, and imaged by microscope (OLYMPUS).

Whole exome sequencing (WES)

Genomic DNA was extracted from parental tissues and urine and tumor CRCs using the TIANamp Genomic DNA Kit (TIANGEN). The exomes were captured using SeqCap EZ MedExome Target Enrichment Kit. Captured DNA fragments were sequenced on an Illumina Novaseq as paired-end 150-bp reads. Reads were aligned to the hg19 version of the human genome using Burrows-Wheeler Aligner software (BWA, version 0.5.9). PCR duplicates were marked using the MarkDuplicates tool in Picard. IndelRealigner and BaseRecalibrator in the Genome Analysis Toolkit (GATK; version 3.8) were used to realign and recalibrate the BWA alignment results. Varscan was used for identifying paired-sample variant calling of SNVs and indels on tumor and matched normal samples. All variants were annotated using Annovar.

To ensure the quality of data, the following criteria were performed to filter raw variant results: average effective sequencing depth on target per sample ≥ 200x; allele mutation frequency ≥ 5% and ≥ 10% for single nucleotide variation and insertion or deletion, respectively; all reads were filtered by high mapping quality (≥ 30) and base quality (≥ 30); and the mutant reads must be supported by positive and negative strands.

hTERT mutation analysis

Genomic DNA of urine CRCs, tumor CRCs, tumor tissues and blood was extracted by TIANamp Genomic DNA Kit following the manufacturer’s instructions (TIANGEN). Primers for mutant hTERT promoter C124T and C146T were

synthesized as 5’-CACCCGTCCTGCCCCTTCACCTT-3’ and 5’-GGCTTCCCACGTGCGCAGCAGGA-3’, respectively (Sangon). These two primers were used to amplify target DNA in 50 μL PCR reactions in 1X high-fidelity hot start DNA polymerase (Biotool) containing 200 ng template. After incubation at 95℃ for 180 seconds, 34 cycles of PCR were performed in the following manner: 95℃ for 30 seconds, 60℃ for 30 seconds, and 72℃ for 30 seconds, and then the final elongation at 72℃ for 5 min. The purified PCR products were sequenced by Sangon Company (Songjiang District, Shanghai, China) and analyzed using Snapgene software.

STR analysis

Total DNA was isolated from urine CRCs, tumor CRCs, tumor tissues，and germline control blood cells using the TIANamp Genomic DNA Kit (TIANGEN). The following STR markers were tested: D8S1179, D21S11, D7S820, CSF1PO, D3S1358, D5S818, D13S317, D16S539, D2S1338, vWA, D12S391, D18S51, Amelogenin, D6S1043, and FGA. Detection of the amplified fragments was performed using the ABI 3100 genetic analyzer (Applied Biosystems). Data analysis were achieved with GeneMapper Software (Applied Biosystems).

Drug sensitivity testing

CRCs derived from urine and bladder tumor tissues were used for the drug response test with 64 clinical oncology drugs, containing chemotherapy drugs and targeted drugs. All drugs were bought from DC Chemicals (Jung-gu, Seoul, Korea) and

Selleck (Houston, Texas, USA). Briefly, cells were plated in 384-well microtiter plates at 400 cells per well in 50 μL conditional medium, and 12 h later, after cell adherence, they were exposed to drugs with series different concentrations for 72 hours. The CRCs' viability was measured with CellTiterGlo (Promega) by Envision plate reader from PerkinElmer.

Statistical analysis

A sigmoidal concentration-response curve was fitted with GraphPad Prism 7 software and half-maximal inhibitory concentration (IC_{50}) was calculated. Drug sensitivity scores (DSS) were calculated by the ratio of each sample to 5637, then applying a log 10 scale. The clustered heatmaps (v. 1.0.10) were then generated by R package pheatmap (v.3.5.1, http://www.r-project.org/).

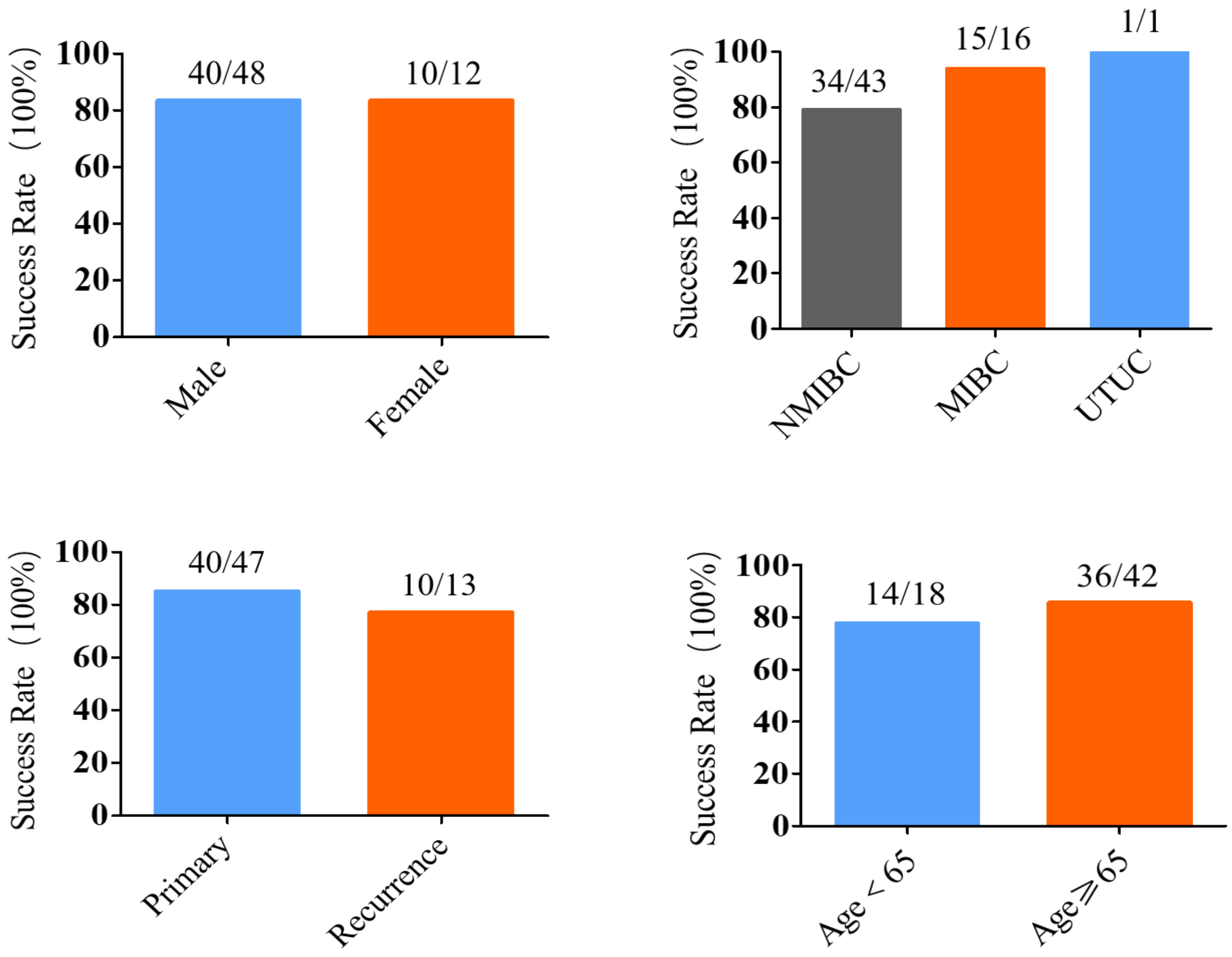

Supplementary Figure S1. Success rate of urine CRCs.

The similar high success rate indicating that CRCs can be established without the bias from all bladder cancer patients.

A

B

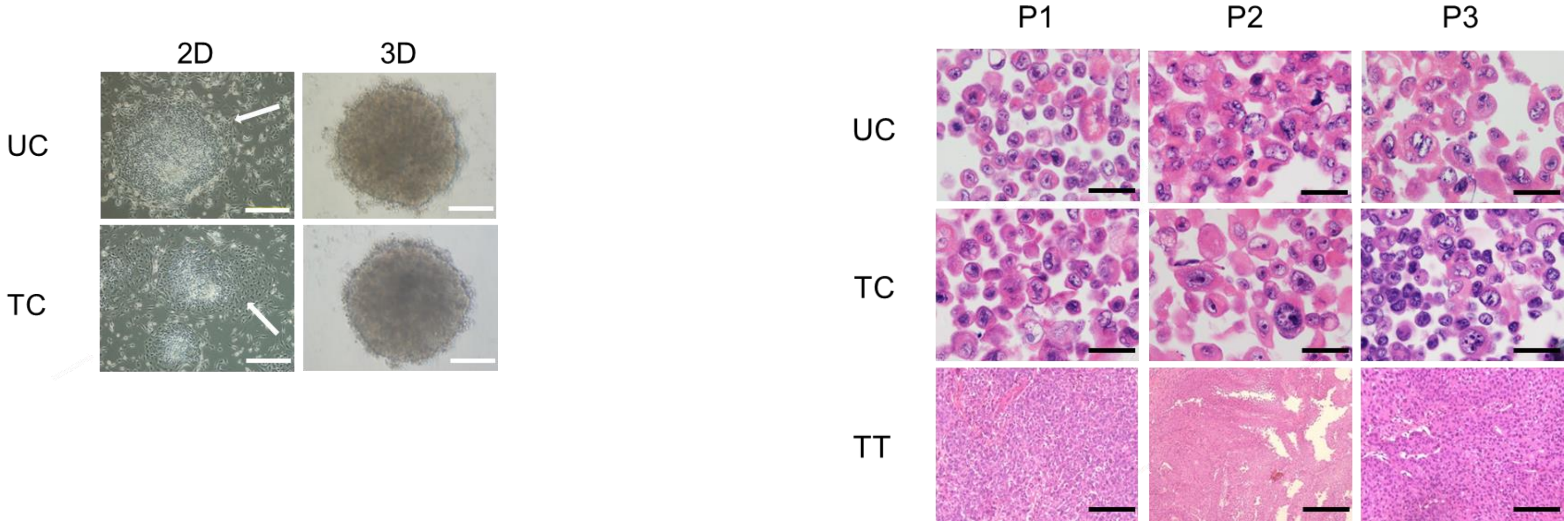

Supplementary Figure S2. Characterization of urine and tumor CRCs.

(A) The light microscope images of 2D and 3D culture of urine and tumor CRCs. The established urine and tumor CRCs all rapidly formed cell colonies and have the ability of spheroid formation (representative image of >40 independent CRCs). The scale bars indicate 500 µm. (B) Representative HE staining of urine and tumor CRCs from patient 1, 2 and 3 and corresponding tumor tissues, used for determining the tumor status and histological origin (representative image of at least n = 3 independent). The scale bars indicate 50 µm (CRCs) and 200 µm (tissues).

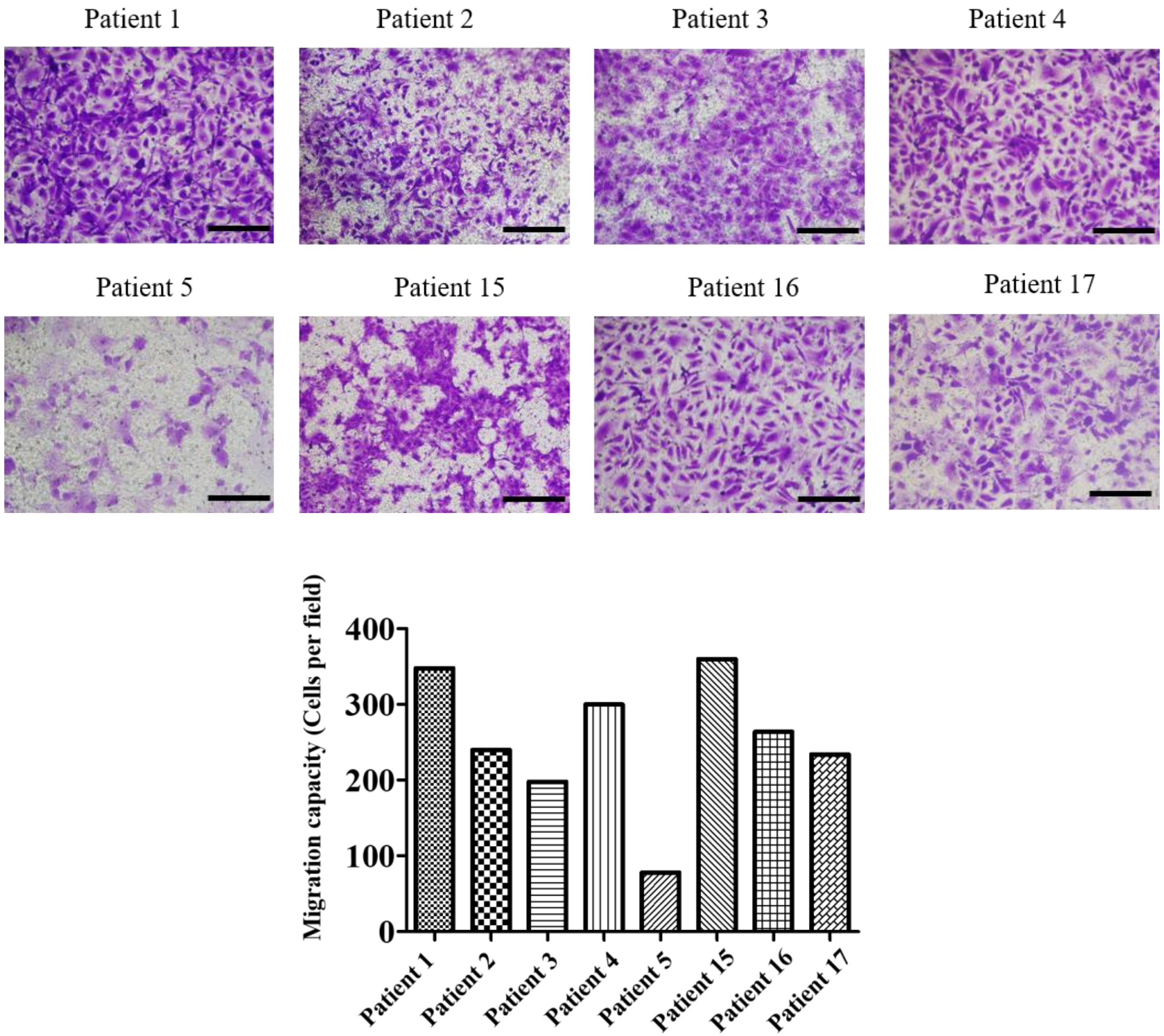

Supplementary Figure S3. Transwell assays of urine CRCs.

Transwell assays of several urine CRCs shows difference of the invasion ability from different patient origin (n = 3 independent experiments). The scale bars indicate 200 μm.

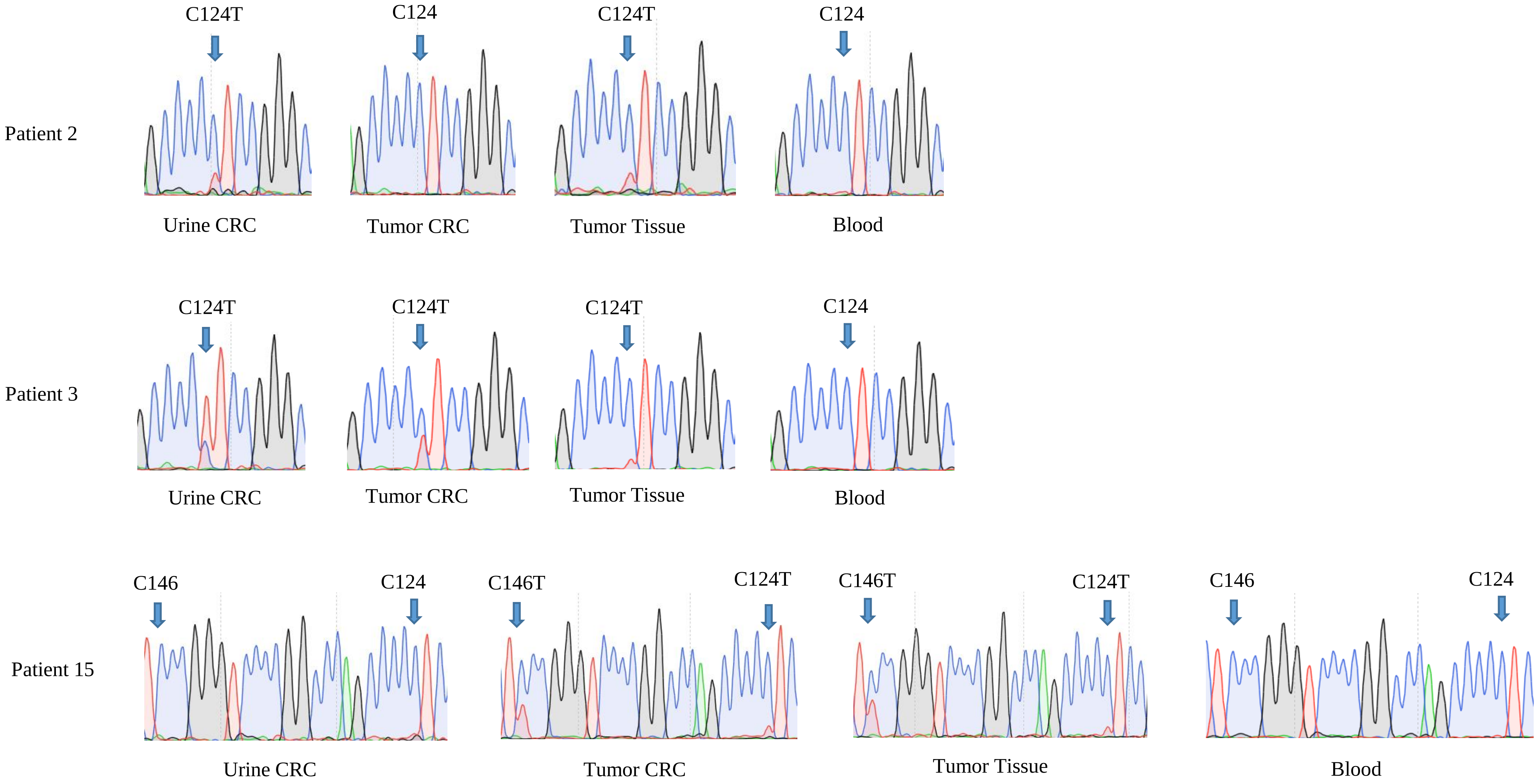

Supplementary Figure S4. Somatic hTERT promoter mutation in bladder cancer tissue, CRCs and blood.

The C124T and C146T mutations are detected in tumor tissues, urine CRCs and tumor CRCs but not in blood samples. hTERT = human telomerase reverse transcriptase.

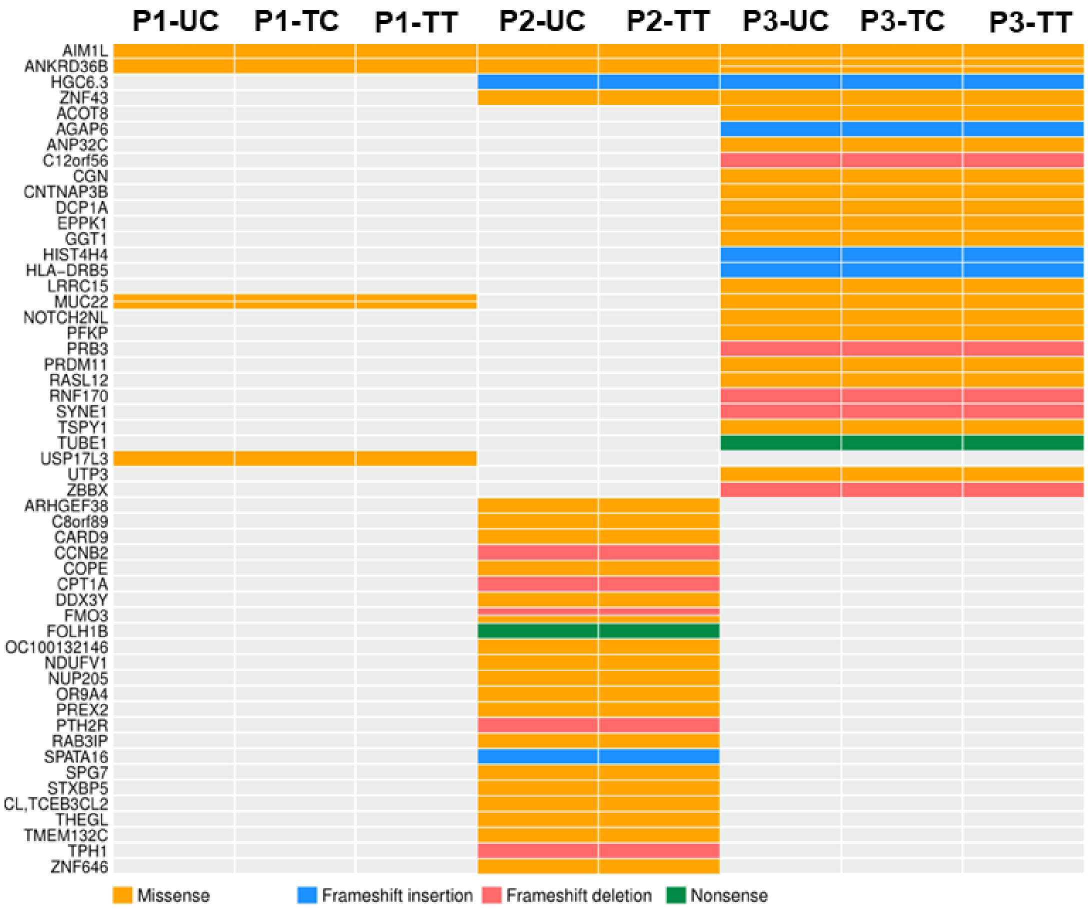
P1-UC
P1-TC
P1-TT
P2-UC
P2-TT
P3-UC
P3-TC
P3-TT
AIM1L
ANKRD36B
HGC6.3
ZNF43
ACOT8
AGAP6
ANP32C
C12orf56
CGN
CNTNAP3B
DCP1A
EPPK1
GGT1
HIST4H4
HLA-DRB5
LRRC15
MUC22
NOTCH2NL
PFKP
PRB3
PRDM11
RASL12
RNF170
SYNE1
TSPY1
TUBE1
USP17L3
UTP3
ZBBX
ARHGEF38
C8orf89
CARD9
CCNB2
COPE
CPT1A
DDX3Y
FMO3
FOLH1B
OC100132146
NDUFV1
NUP205
OR9A4
PREX2
PTH2R
RAB3IP
SPATA16
SPG7
STXBP5
CL,TCEB3CL2
THEGL
TMEM132C
TPH1
ZNF646
Missense
Frameshift insertion
Frameshift deletion
Nonsense

Supplementary Figure S5. Exome sequence in urine and tumor CRCs.

Landscape of common somatic mutations among paired urine CRCs, tumor CRCs and tumor tissues for the same patient. Samples are arranged in columns, genes are arranged in rows. Mutation types are displayed by different colors in the bottom panel.

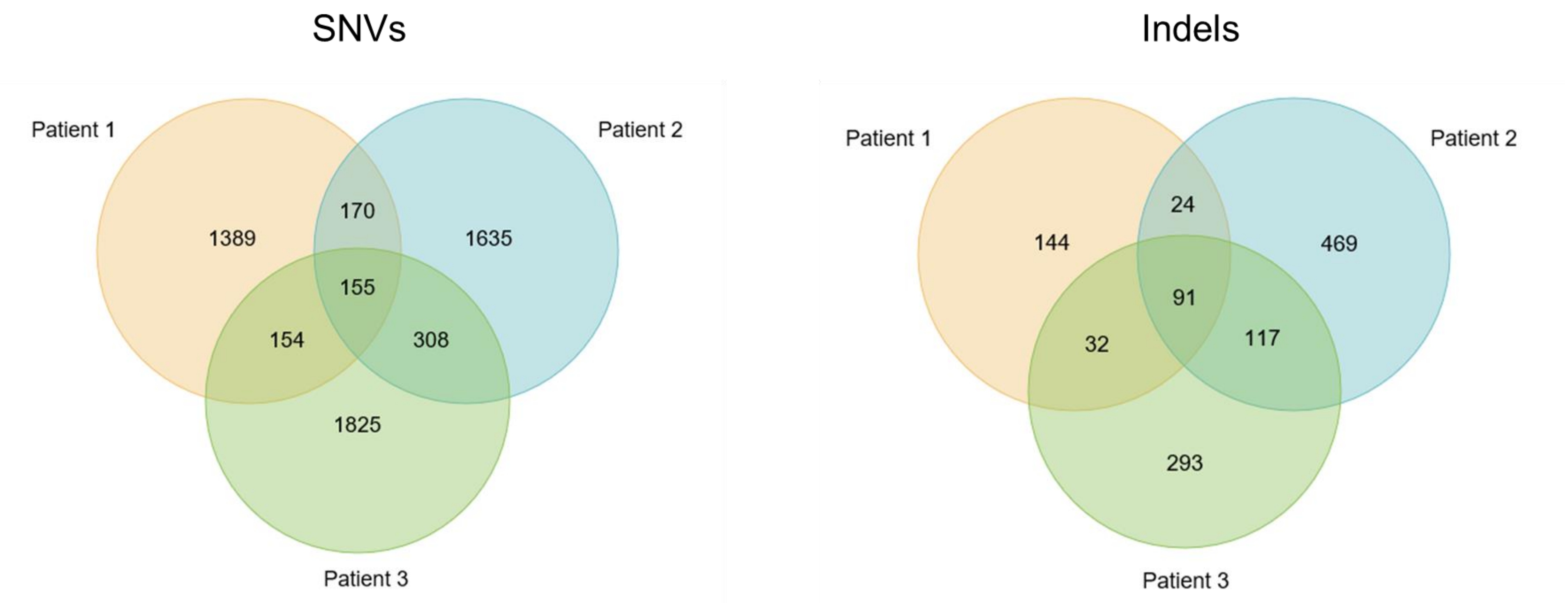

Supplementary Figure S6. SNVs and Indels in tumor tissues of Patient 1, 2, 3.

Venn diagram of SNVs and Indels in formalin-fixed paraffin-embedded samples of patient 1, 2 and 3. Mutation numbers are showed within the circles.

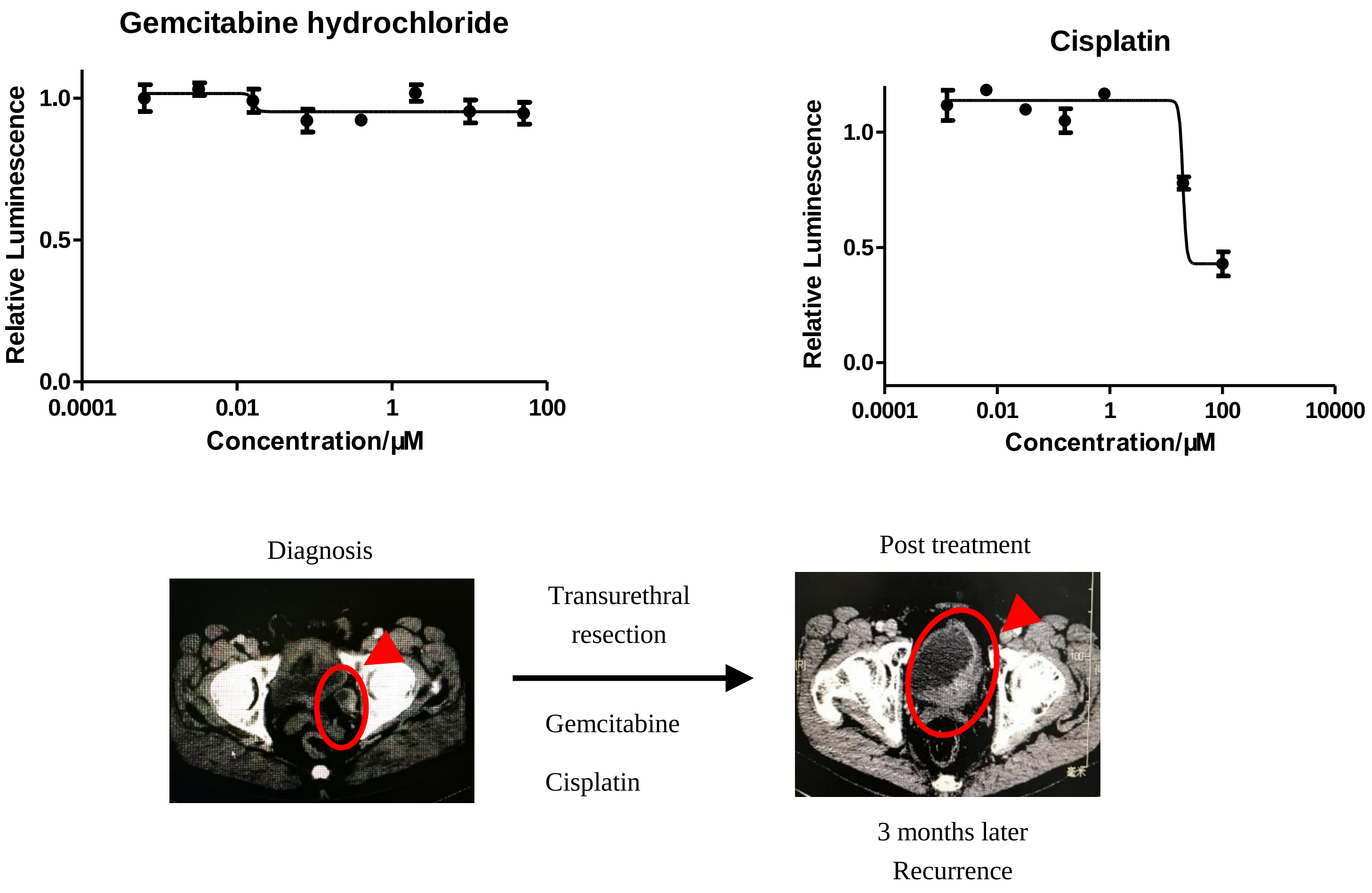

Supplementary Figure S7. Drug sensitivity and corresponding clinical relevance of urine CRC from patient 21.

Urine CRC's dose-response curves for the indicated drugs are exhibited in the upper row, and the CT of the patient before and after treatment are shown in the lower row. Urine CRCs isolated from patient 21 showed resistance to gemcitabine and low sensitivity to cisplatin, which is closely related to the prognosis of patient 21 who accepted tumor resection followed chemotherapy but relapse soon. Red circles and arrows indicate primary or recurrence tumors.

Supplementary Table S1. Patient characteristics and corresponding conditionally reprogrammed cells (CRC)

	Male/ Female	Age	primary/ recurrence	pathology	TNM	NMIBC/ MIBC	Tumor number	Risk group	treatment	Tumor CRC	Mutation of hTERT promoter	Urine CRC	Mutation of hTERT promoter
Patient 1	F	83	p	H	T3N0M0	MIBC	s	H	PC+Epirubicin.	1	no	1	no
Patient 2	M	57	p	H	TaN0M0	NMIBC	s	H	TURBT+Pirarubicin	1	no	1	yes
Patient 3	M	85	r	H	T1N0M0	NMIBC	m	H	TURBT+Pirarubicin	1	yes	1	yes
Patient 4	M	63	p	H	TaN0M0	NMIBC	m	H	RC+Epirubicin	1	yes	1	yes
Patient 5	M	80	r	H	T4N1M1	MIBC	m	H	Gemcitabine+Cisplatin	1	yes	1	yes
Patient 6	M	68	p	H	T2N0M0	MIBC	m	H	RC	1	ND	1	ND
Patient 7	M	60	p	H	T1N0M0	NMIBC	m	H	TURBT+Gemcitabine+Cisplatin+Epirubicin.	-		1	ND
Patient 8	M	87	r	H	T3N3M0	MIBC	m	H	Gemcitabine+Cisplatin	-		1	ND
Patient 9	M	74	r	H	TaN0M0	NMIBC	m	H	TURBT+Epirubicin+BCG	-		1	ND
Patient 10	M	79	p	H	T2N0M0	MIBC	m	H	RC	1	ND	1	ND
Patient 11	F	52	p	H	TaN0M0	NMIBC	m	H	TURBT+Epirubicin	-		1	ND
Patient 12	M	48	p	H	T2N0M0	MIBC	m	H	RC	1	ND	1	ND
Patient 13	M	62	r	H	T4N0M0	MIBC	m	H	TURBT+Gemcitabine	-		1	ND
Patient 14	M	62	p	H	T3N0M0	MIBC	m	H	RC	1	yes	1	yes
Patient 15	F	79	p	H	T1N0M0	NMIBC	m	H	TURBT+Pirarubicin	1	yes	1	no
Patient 16	M	82	p	H	T1N0M0	NMIBC	m	H	TURBT+Epirubicin	1	yes	1	no
Patient 17	F	62	p	H	T1N0M0	NMIBC	m	H	TURBT	1	no	1	no
Patient 18	F	61	p	L	TaN0M0	NMIBC	s	L	TURBT	0		1	ND
Patient 19	M	69	p	L	T1N0M0	NMIBC	m	H	RC	-		1	ND
Patient 20	F	83	r	H	T1N0M0	NMIBC	m	H	TURBT+Epirubicin	1	no	1	no
Patient 21	M	75	p	H	T3N0M0	MIBC	m	H	TURBT+ Gemcitabine+Cisplatin	-		1	ND
Patient 22	M	68	p	H	T1N0M0	NMIBC	s	H	TURBT+Epirubicin	1	ND	1	ND
Patient 23	M	56	p	H	T1N0M0	NMIBC	m	H	TURBT+Epirubicin	1	yes	1	yes
Patient 24	M	78	p	H	T2N0M0	NMIBC	m	H	TURBT+Pirarubicin	1	no	1	no
Patient 25	M	80	r	L	TaN0M0	NMIBC	m	H	TURBT+Pirarubicin	-		1	ND
Patient 26	M	87	p	H	T1N0M0	NMIBC	m	H	TURBT+Pirarubicin	-		1	ND
Patient 27	M	88	r	H	T1N0M0	NMIBC	m	H	TURBT	0		1	ND
Patient 28	M	73	p	H	TaN0M0	NMIBC	m	H	TURBT+Epirubicin	1	ND	1	ND

Patient 29	M	55	p	H	T1N0M0	NMIBC	m	H	TURBT+Epirubicin	1	ND	1	ND
Patient 30	M	79	p	H	T1N0M0	NMIBC	m	H	TURBT+Epirubicin+BCG	1	ND	1	ND
Patient 31	M	79	p	H	T2N0M1	MIBC	m	H	RC+Gemcitabine+Cisplatin	-		1	ND
Patient 32	M	80	p	H	T4N1M1	UTUC	s	H	Oxaliplatin+Pemetrexe	-		1	ND
Patient 33	M	69	p	H	T1N0M0	NMIBC	m	H	TURBT+Pirarubicin	0		1	ND
Patient 34	F	78	p	L	TaN0M0	MIBC	m	H	TURBT+Epirubicin	-		1	ND
Patient 35	M	63	r	H	T3N0M0	MIBC	m	H	Durvalumab+CTLA-4+TURBT	-		1	ND
Patient 36	M	73	p	H	T1N0M0	NMIBC	m	H	TURBT+Epirubicin	1	ND	0	
Patient 37	F	71	r	H	T1N0M0	NMIBC	s	H	TURBT+Epirubicin	-		0	
Patient 38	M	67	p	H	T3N0M0	MIBC	m	H	RC	-		0	
Patient 39	M	66	r	H	T1N0M0	NMIBC	s	H	TURBT+BCG	-		0	
Patient 40	M	33	p	L	T1N0M0	NMIBC	s	L	TURBT+Pirarubicin	-		0	
Patient 41	F	61	p	H	T1N0M0	NMIBC	s	H	TURBT+Pirarubicin	-		0	
Patient 42	M	87	p	H	T1N0M0	NMIBC	m	H	TURBT+Epirubicin	-		0	
Patient 43	M	69	p	L	T1N0M0	NMIBC	s	L	TURBT+Pirarubicin	1	ND	1	ND
Patient 44	M	62	p	H	T2N0M0	MIBC	m	H	RC	1	ND	1	ND
Patient 45	F	66	p	H	T1N0M0	NMIBC	m	H	TURBT	1	ND	1	ND
Patient 46	M	61	p	L	T1N0M0	NMIBC	m	H	TURBT	1	ND	0	
Patient 47	M	86	P	L	T1N0M0	NMIBC	s	L	TURBT	-		1	ND
Patient 48	M	86	P	L	T1N0M0	NMIBC	s	L	TURBT	1	ND	1	ND
Patient 49	M	69	P	H	T1N0M0	NMIBC	m	H	RC	-		1	ND
Patient 50	M	58	p	L	T1N0M0	NMIBC	m	H	PC+Epirubicin	1	ND	0	
Patient 51	F	85	p	H	T1N0M0	NMIBC	m	H	lost	1	ND	1	ND
Patient 52	F	75	p	H	T1N0M0	NMIBC	m	H	TURBT+Pirarubicin	-		1	ND
Patient 53	M	71	p	H	T1N0M0	NMIBC	m	H	TURBT+Epirubicin	1	ND	1	ND
Patient 54	M	84	p	H	T3N0M0	MIBC	s	H	RC	-		1	ND
Patient 55	M	68	r	L	T1N0M0	NMIBC	s	H	TURBT+Pirarubicin	-		1	ND
Patient 56	M	51	r	L	T1N0M0	NMIBC	m	H	TURBT+Pirarubicin	-		1	ND
Patient 57	M	69	p	H	T1N0M0	NMIBC	m	H	TURBT+Pirarubicin	-		0	
Patient 58	M	67	p	H	T1N0M0	NMIBC	s	H	TURBT+Epirubicin	1	ND	1	ND
Patient 59	M	79	p	H	T1N0M0	NMIBC	m	H	TURBT+Epirubicin	1	ND	1	ND
Patient 60	M	75	p	H	T3N0M0	MIBC	m	H	RC	-		1	ND

60 patients were enrolled and been classified by gender, age, pathology, tumor number, TNM and risk group. Pathology: H=high grade, L=low grade; MIBC=Muscle-invasive bladder cancer, NMIBC=Non-muscle-invasive bladder cancer, UTUC= Upper tract urothelial carcinoma; Tumor number: s=single, m=multiple; Risk group: H=high risk, L=low risk; Treatment: TURBT=transurethral resection of bladder tumor, PC=partial cystectomy, RC=radical cystectomy. Tumor/urine CRC: 1=successfully established, 0= fail to establish, - = No sample, ND = No detect.

Supplementary Table S2. STR analysis of bladder cancer tissues, CRCs and blood

Samples		STR Alleles															
		D8S1179	D21S11	D7S820	CSF1PO	D3S1358	D5S818	D13S317	D16S539	D2S1338	D19S433	vWA	D12S391	D18S51	Amelogenin	D6S1043	FGA
Patient 1	Tumor Tissue	11 , 13	29	10 , 11	10 , 12	16 , 18	12	8 , 9	9 , 11	20 , 24	13 , 15.2	14 , 16	15 , 19	15	X	13 , 20	22 , 23
	Tumor CRC	11 , 13	29	10 , 11	10 , 12	16	12	8 , 9	9 , 11	20 , 24	13 , 15.2	14 , 16	15 , 19	15	X	13 , 20	22
	Urine CRC	11 , 13	29	10 , 11	10 , 12	16	12	8 , 9	9 , 11	20 , 24	13 , 15.2	14 , 16	15 , 19	15	X	13 , 20	22
	Blood	11 , 13	29	10 , 11	10 , 12	16 , 18	12	8 , 9	9 , 11	20 , 24	13 , 15.2	14 , 16	15 , 19	15	X	13 , 20	22 , 23
Patient 2	Tumor Tissue	14	30 , 33	11 , 13	10 , 11	16 , 17	11	11 , 12	11 , 12	21 , 24	13	14 , 16	18 , 19	13 , 14	X , Y	15 , 18	19 , 24
	Tumor CRC	14	30 , 33	11 , 13	10 , 11	16 , 17	11	11 , 12	11 , 12	21 , 24	13	14 , 16	18 , 19	13 , 14	X , Y	15 , 18	19 , 24
	Urine CRC	14	30 , 33	11 , 13	10 , 11	16 , 17	11	11 , 12	11 , 12	21 , 24	13	14 , 16	18 , 19	13 , 14	X , Y	15 , 18	19 , 24
	Blood	14	30 , 33	11 , 13	10 , 11	16 , 17	11	11 , 12	11 , 12	21 , 24	13	14 , 16	18 , 19	13 , 14	X , Y	15 , 18	19 , 24
Patient 3	Tumor Tissue	16	32 , 32.2	11	9 , 10	15 , 16	10	10 , 11	11 , 12	24 , 26	13 , 15.2	16 , 17	18 , 19	15 , 16	X , Y	14 , 18	23 , 24
	Tumor CRC	16	32 , 32.2	11	9 , 10	15 , 16	10	10 , 11	11 , 12	24	13 , 15.2	16 , 17	18 , 19	15 , 16	X , Y	14 , 18	23 , 24
	Urine CRC	16	32 , 32.2	11	9 , 10	15 , 16	10	10 , 11	11 , 12	24	13 , 15.2	16 , 17	18 , 19	15 , 16	X , Y	14 , 18	23 , 24
	Blood	16	32 , 32.2	11	9 , 10	15 , 16	10	10 , 11	11 , 12	24 , 26	13 , 15.2	16 , 17	18 , 19	15 , 16	X , Y	14 , 18	23 , 24

**Patient 4**	**Tumor Tissue**	10, 11	32, 32.2	11, 12	10, 12	15, 18	8, 11	8, 12	9	20, 24	13, 15.2	13, 16	17, 19	13, 15	X, Y	12, 18	19, 25
	**Tumor CRC**	10, 11	32, 32.2	11, 12	10, 12	15, 18	8, 11	8, 12	9	20, 24	13, 15.2	13, 16	17, 19	13, 15	X, Y	12, 18	19, 25
	**Urine CRC**	10, 11	32, 32.2	11, 12	10, 12	15, 18	8, 11	8, 12	9	20, 24	13, 15.2	13, 16	17, 19	13, 15	X, Y	12, 18	19, 25
	**Blood**	10, 11	32, 32.2	11, 12	10, 12	15, 18	8, 11	8, 12	9	20, 24	13, 15.2	13, 16	17, 19	13, 15	X, Y	12, 18	19, 25
**Patient 5**	**Tumor Tissue**	12 , 16	28 , 29	8 , 11	10 , 13	16	8 , 10	9 , 11	9 , 12	20 , 21	13	18	18 , 22	13 , 17	X , Y	11 , 20	23 , 24
	**Tumor CRC**	12 , 16	28 , 29	8 , 11	10 , 13	16	8 , 10	9 , 11	9 , 12	20 , 21	13	18	18 , 22	13	X , Y	11 , 20	23 , 24
	**Urine CRC**	12 , 16	28 , 29	8 , 11	10 , 13	16	8 , 10	9 , 11	9 , 12	20 , 21	13	18	18 , 22	13 , 17	X , Y	11 , 20	23 , 24
	**Blood**	12 , 16	28 , 29	8 , 11	10 , 13	16	8 , 10	9 , 11	9 , 12	20 , 21	13	18	18 , 22	13 , 17	X , Y	11 , 20	23 , 24
**Patient 14**	**Tumor Tissue**	12 , 13	29 , 30	10 , 12	9 , 10	15 , 16	10 , 13	9 , 12	9 , 13	20 , 25	13 , 13.2	16 , 20	18 , 20	13 , 19	X , Y	19	22 , 24
	**Tumor CRC**	12 , 13	29 , 30	10 , 12	9 , 10	15 , 16	10 , 13	9 , 12	9 , 13	20 , 25	13 , 13.2	16 , 20	18 , 20	13 , 19	X , Y	19	22 , 24
	**Urine CRC**	12 , 13	29 , 30	10 , 12	9 , 10	15 , 16	10 , 13	9 , 12	9 , 13	20 , 25	13 , 13.2	16 , 20	18 , 20	13 , 19	X , Y	14 , 19	22 , 24
	**Blood**	12 , 13	29 , 30	10 , 12	9 , 10	15 , 16	10 , 13	9 , 12	9 , 13	20 , 25	13 , 13.2	16 , 20	18 , 20	13 , 19	X , Y	14 , 19	22 , 24
**Patient 15**	**Tumor Tissue**	12 , 16	31 , 31.2	11 , 12	11 , 12	17	11	9 , 13	9 , 12	18	14 , 16	20 , 21	19 , 20	14 , 15	X , Y	19 , 20	23
	**Tumor CRC**	12 , 16	31 , 31.2	11 , 12	11 , 12	17	11	9 , 13	9 , 12	18	14 , 16	20 , 21 , 22	19 , 20	14 , 15	X , Y	19 , 20	23

	Urine CRC	12 , 16	31 , 31.2	11 , 12	11 , 12	17	11	9 , 13	9 , 12	18	14 , 16	20 , 21	19 , 20	14 , 15	X , Y	19 , 20	23
	Blood	12 , 16	31 , 31.2	11 , 12	11 , 12	17	11	9 , 13	9 , 12	18	14 , 16	20 , 21	19 , 20	14 , 15	X , Y	19 , 20	23
Patient 16	Tumor Tissue	10 , 11	28 , 29	11 , 13	12	15 , 16	9 , 10	11 , 12	11	17 , 22	13 , 14.2	16 , 19	18 , 19	13	X	11 , 19	25 , 26
	Tumor CRC	10 , 11	28 , 29	11 , 13	12	15 , 16	9 , 10	11 , 12	11	17 , 22	13 , 14.2	16 , 19	18 , 19	13	X	11 , 19	25 , 26
	Urine CRC	10 , 11	28 , 29	11 , 13	12	15 , 16	9 , 10	11 , 12	11	17 , 22	13 , 14.2	16 , 19	18 , 19	13	X	11 , 19	25 , 26
	Blood	10 , 11	28 , 29	11 , 13	12	15 , 16	9 , 10	11 , 12	11	17 , 22	13 , 14.2	16 , 19	18 , 19	13	X	11 , 19	25 , 26
Patient 17	Tumor Tissue	10 , 12	29 , 34.2	11 , 12	11 , 12	15 , 17	9 , 11	8 , 13	11 , 13	23 , 24	13	18	19 , 22	15 , 21	X , Y	12 , 18	24
	Tumor CRC	10 , 12	29 , 34.2	11 , 12	11 , 12	15 , 17	9 , 11	8 , 13	11 , 13	23 , 24	13	18	19 , 22	15 , 21	X , Y	12 , 18	24
	Urine CRC	10 , 12	29 , 34.2	11 , 12	11 , 12	15 , 17	9 , 11	8 , 12	11 , 13	23 , 24	13	18	19 , 22	15 , 21 , 23	X , Y	12 , 18	24
	Blood	10 , 12	29 , 34.2	11 , 12	11 , 12	15 , 17	9 , 11	8 , 13	11 , 13	23 , 24	13	18	19 , 22	15 , 21	X , Y	12 , 18	24

Supplementary Table S3. STR analysis of early- and late- passage bladder cancer urine CRCs

Samples	STR Alleles															
	D8S1179	D21S11	D7S820	CSF1PO	D3S1358	D5S818	D13S317	D16S539	D2S1338	D19S433	vWA	D12S391	D18S51	Amelogenin	D6S1043	FGA
Patient 1 urine CRC P1	11, 13	29	10, 11	10,12	16	12	8, 9	9, 11	20, 24	13, 15.2	14, 16	15, 19	15	X	13, 20	22
Patient 1 urine CRC P19	11, 13	29	10, 11	10,12	16	12	8, 9	9, 11	20, 24	13, 15.2	14, 16	15, 19	15	X	13, 20	22

The results of DNA fingerprinting of passage 1 and 19 urine CRCs in patient 1 demonstrated that they have 15 identical STR loci and the X-specific Amelogenin locus, thereby verifying their genetic stability.

Supplementary Table S4. Mutations of hTERT promoter in bladder cancer tissues and CRCs

hTERT promoter mutation	C124T	C146T
Tumor Tissue	8/12 (66.7 %)	3/12(25.0 %)
Tumor CRCs	7/12 (58.3 %)	2/12 (16.7 %)
Urine CRCs	6/12 (50.0 %)	0/12 (0 %)
Blood	0/12 (0 %)	0/12 (0 %)

Overall distribution of hTERT promoter mutations in urine CRCs, tumor CRCs, tumor tissues and blood cells.

Supplementary Table S5. The IC_{50} and drug sensitivity scores (DSS) of CRCs

The IC_{50} of CRCs（μM)

Drugs	P1-UC	P1-TC	P2-UC	P3-UC	P3-TC	P4-UC	P4-TC	P5-UC	P6-UC	P6-TC	P7-UC	P8-UC	P9-UC	P10-UC	P11-UC	P12-UC	P13-UC	5637
**Temsirolimus**	Over	Over	Over	Over	Over	Over	Over	Over	Over	Over	Over	Over	Over	Over	Over	Over	0.0021159	0.0008468
**Imatinib**	Over	Over	Over	Over	Over	Over	Over	Over	Over	Over	Over	Over	Over	Over	Over	Over	0.0019712	Over
**Regorafenib**	26.714631	Over	Over	Over	Over	Over	Over	Over	Over	Over	Over	Over	Over	Over	Over	Over	Over	Over
**Sorafenib Mesylate**	5.4672527	4.6486556	16.574713	Over	2.6308364	45.154291	0.9206483	3.7667775	Over	Over	Over	Over	Over	10.723775	7.8440287	10.184582	0.0304903	Over
**Palbociclib**	Over	Over	Over	Over	Over	Over	Over	Over	Over	Over	Over	Over	Over	Over	Over	Over	0.0032174	Over
**Ponatinib**	0.0108067	0.0208055	0.5866431	0.1193234	0.0951992	1.0412263	Over	0.0275922	Over	Over	0.9946389	0.9713251	Over	0.9926649	0.1476521	0.0766279	0.0013395	0.1105454
**Vinorelbine**	0.0001360	Over	Over	Over	0.0050704	Over	Over	0.0001091	Over	Over	Over	0.0034912	Over	Over	Over	Over	0.0013306	0.0078498
**Cyclophosphamide**	Over	Over	Over	Over	Over	Over	Over	Over	Over	Over	Over	Over	Over	Over	Over	Over	Over	Over
**Ifosfamide**	Over	Over	Over	Over	Over	Over	Over	Over	Over	Over	Over	Over	Over	Over	Over	Over	0.0013755	Over
**Daunorubicin**	0.0318938	0.0377498	0.0859899	0.0902950	0.0533235	0.1488192	0.0958124	0.0526311	0.2335186	0.3199119	0.0844582	0.0326209	Over	0.1134617	0.0266141	0.0440041	0.0057504	0.0121611
**Idarubicin**	0.0612663	0.0444453	0.8139187	0.1565709	0.0296415	14.582804	0.0842599	0.0769727	0.4037414	0.4336732	0.0831817	0.0309445	0.1306952	0.1760055	0.0467046	0.1607318	0.0004997	1.1602087
**Irinotecan**	51.709652	Over	Over	Over	16.395090	Over	Over	Over	Over	Over	5.7008091	9.5656105	13.421787	Over	Over	12.069299	Over	9.6322439
**Anastrozole**	Over	Over	Over	Over	Over	Over	Over	Over	Over	Over	Over	Over	Over	Over	Over	Over	Over	Over
**Tamoxifen**	1.5595892	1.2906039	11.631802	3.0080787	Over	11.451936	11.7	1.2586674	Over	Over	111.37395	1.1220919	Over	Over	11.526841	Over	Over	Over
**Etoposide**	Over	Over	Over	Over	Over	Over	Over	Over	Over	Over	0.0866244	0.9899651	Over	Over	Over	Over	Over	11.859265
**Mercaptopurine**	Over	Over	Over	Over	Over	Over	Over	Over	Over	Over	Over	Over	Over	Over	Over	Over	Over	Over
**Sirolimus**	Over	Over	Over	Over	14.236765	Over	Over	Over	Over	Over	Over	Over	Over	Over	Over	Over	Over	Over
**Exemestane**	11.724301	24.083542	Over	Over	Over	Over	Over	Over	Over	Over	Over	Over	Over	Over	Over	Over	Over	Over
**Mitoxantrone**	0.0810884	0.0513711	0.1684946	0.1510885	0.0350101	0.1987783	0.1217035	0.0705499	0.1858976	0.25436	3.2348034	Over	1.2146787	0.1731317	0.0522750	0.1345354	Over	0.2458160
**Cladribine**	Over	Over	Over	Over	3.2119843	Over	Over	Over	Over	Over	Over	Over	Over	Over	Over	Over	Over	Over
**Cytarabine**	Over	Over	Over	Over	5.3704745	Over	Over	Over	Over	1.6598182	5.3831100	Over	Over	Over	Over	Over	Over	Over
**Hydroxyurea**	Over	Over	Over	Over	Over	Over	Over	Over	Over	Over	Over	Over	Over	Over	Over	Over	Over	Over
**Gemcitabine Hydrochloride**	Over	0.0108536	Over	Over	0.0203047	Over	Over	0.005581	Over	Over	0.007195	0.0068431	Over	Over	Over	Over	Over	0.0099081
**Cisplatin**	12.981416	Over	7.8976457	44.007304	18.287253	Over	Over	8.172	Over	Over	4.0565837	Over	44.002538	Over	Over	31.637134	Over	2.772
**Carboplatin**	Over	Over	Over	46.836621	Over	Over	Over	29.93	Over	Over	Over	Over	Over	Over	Over	Over	Over	Over
**Nedaplatin**	9.0288126	5.3392940	6.4412637	Over	Over	Over	6.8385986	25.47	Over	Over	Over	Over	Over	6.1006219	16.438103	0.8408679	Over	4.674
**Methotrexate**	Over	Over	Over	Over	Over	Over	Over	Over	Over	Over	Over	Over	Over	Over	Over	Over	Over	Over
**Vinblastine sulfate**	Over	Over	Over	Over	0.0003688	Over	Over	0.0006912	Over	Over	Over	0.0023061	Over	Over	Over	Over	Over	0.0082349
**Doxorubicin Hydrochloride**	0.0166376	0.0257598	0.0482377	0.0204724	0.0088632	0.0590113	0.0267613	0.0139995	0.4371304	0.7188709	0.0209881	0.0068714	0.0313215	0.0445547	0.0267132	0.0178623	Over	0.0861818

**Epirubicin Hydrochloride**	0.0200869	0.0637246	0.0756127	0.1592866	3.15551E-	0.1426366	0.0864943	0.0691333	1.3069702	1.0776303	0.2708869	Over	1.0256234	0.1293203	0.1237359	0.1617529	Over	0.0751392
**Pemetrexed**	Over	Over	Over	Over	Over	Over	Over	Over	Over	Over	Over	Over	Over	Over	Over	Over	Over	0.008708
**Pirarubicin**	5.0125063	Over	11.484713	11.894037	1.6714404	1.3127984	1.0859436	2.872	Over	Over	Over	Over	Over	1.1127987	Over	1.2924315	Over	0.78
**Hydeoxycamptothecin**	0.1006173	0.2079719	Over	1.1016199	0.0674857	Over	7.0986096	0.01537	Over	Over	Over	Over	Over	Over	0.3691486	1.0156460	Over	0.0669329
**Mitomycin**	1.1776134	11.570252	Over	Over	2.0338478	Over	Over	0.006438	0.8611372	1.0556795	1.8943340	Over	Over	1.2327655	Over	2.1427471	Over	0.0070137
**Sunitinib**	1.2345394	1.3287155	1.0261644	Over	1.1099789	20.949861	10.747782	5.5685060	Over	Over	1.2139602	Over	Over	Over	Over	Over	Over	10.26
**Everolimus**	Over	Over	Over	Over	Over	Over	Over	12.696347	Over	Over	0.7621397	Over	Over	Over	Over	Over	Over	Over
**Paclitaxel**	Over	Over	Over	Over	Over	Over	Over	0.0005542	Over	Over	0.1426310	0.0001018	Over	Over	Over	Over	Over	0.03369
**Docetaxel**	Over	Over	Over	Over	Over	Over	Over	0.0001188	Over	Over	Over	1.13509E-	Over	Over	Over	Over	Over	0.00658
**Olaparib**	Over	Over	Over	Over	Over	Over	Over	Over	Over	Over	Over	Over	Over	Over	Over	Over	Over	Over
**Erlotinib**	Over	Over	Over	0.3236289	Over	Over	Over	Over	Over	Over	Over	Over	Over	Over	Over	Over	Over	0.0022133
**Afatinib**	Over	Over	1141.2183	Over	0.8313371	Over	0.9957446	1.2666812	Over	Over	Over	Over	0.0015364	Over	Over	Over	Over	0.0014566
**Crizotinib**	5.0729888	Over	1.2415735	1.6333669	0.7081875	1.1328848	0.9757011	1.2140096	Over	1.4209461	1.0902526	Over	Over	1.3036119	Over	1.1704457	Over	0.5004954
**Vemurafenib**	Over	Over	Over	Over	Over	Over	Over	Over	Over	Over	Over	Over	Over	Over	Over	Over	Over	Over
**Trametinib**	Over	Over	Over	Over	Over	Over	0.0126323	Over	Over	0.0004347	Over	Over	Over	Over	Over	Over	Over	Over
**Belinostat**	0.2466097	0.1419838	0.4948366	3.5207720	0.3073521	2.5788110	1.1162556	0.6810221	1.8975196	1.3610460	0.6596448	0.5150037	1.0996778	3.2862910	0.3763404	1.6867041	Over	0.2634739
**Bleomycin**	Over	Over	Over	2.0572426	Over	Over	Over	Over	Over	Over	Over	Over	1.3397909	Over	Over	Over	Over	0.0470373
**Capecitabine**	0.4777202	Over	Over	11.100363	Over	Over	Over	1.1575231	Over	Over	Over	1.0470716	Over	Over	Over	Over	Over	0.7193337
**Doxifluridine**	Over	Over	Over	Over	Over	Over	Over	Over	Over	Over	Over	Over	Over	Over	Over	Over	Over	Over
**Oxaliplatin**	Over	Over	Over	Over	Over	Over	Over	Over	Over	Over	Over	Over	Over	Over	Over	Over	Over	Over
**Floxuridine**	Over	Over	Over	Over	Over	Over	Over	Over	Over	Over	Over	Over	Over	Over	Over	Over	Over	0.8471396
**Fludarabine**	0.0494845	0.0264617	Over	1.0298751	Over	Over	Over	Over	0.0772846	0.0955143	Over	Over	Over	Over	Over	Over	Over	0.1440036
**Dacarbazine**	Over	Over	Over	13.905748	Over	Over	Over	Over	Over	Over	Over	Over	Over	Over	Over	Over	Over	3.6002285
**Topotecan**	Over	Over	0.2566817	4.0227902	2.2048211	0.1402485	Over	Over	Over	1.7729330	0.0396088	0.1246842	Over	Over	Over	6.9104472	Over	11.643994
**Axitinib**	Over	Over	Over	Over	2.2116386	Over	Over	Over	Over	Over	Over	Over	Over	Over	Over	Over	Over	1.9346381
**Bortezomib**	0.0003945	0.0005220	0.0001096	0.0008200	0.0013192	0.0023309	0.0012766	0.0008857	0.0009973	0.0011616	0.0014046	0.0018245	0.0002811	0.0007120	0.0009687	0.0007812	0.0009592	0.0079804
**Dasatinib**	0.0528168	0.0454356	0.1581124	Over	0.0241146	Over	Over	0.0118671	Over	Over	0.0489388	0.0049101	Over	Over	0.0250006	Over	Over	0.0103875
**Gefitinib**	3.5272508	Over	Over	Over	1.7616580	Over	Over	1.8072495	Over	Over	Over	0.0029913	Over	Over	Over	Over	Over	Over
**Ibrutinib**	Over	Over	Over	Over	Over	Over	Over	Over	Over	Over	Over	Over	Over	Over	Over	Over	Over	0.0279229
**Lapatinib**	Over	Over	2.1326666	Over	1.6925159	Over	Over	Over	Over	Over	0.9186711	0.3360162	5.02379E-	Over	Over	Over	Over	0.0101814
**Nilotinib**	3.1531622	11.830530	Over	11.881050	1.1584903	Over	Over	1.2083434	Over	Over	Over	Over	Over	Over	Over	1.4314615	Over	Over

**Pazopanib**	Over	Over	Over	Over	Over	Over	0.1146452	Over	Over	Over	1.3502632	Over	Over	Over	Over	Over	Over	Over
**Trametinib**	Over	Over	Over	Over	Over	Over	Over	Over	Over	Over	Over	Over	Over	Over	Over	Over	Over	0.0014170
**Dabrafenib**	Over	Over	Over	Over	Over	Over	Over	Over	Over	Over	0.56411	Over	Over	Over	Over	Over	Over	0.0462286
**Vorinostat**	0.5614517	0.5633676	2.5760326	1.4909612	0.5397094	Over	Over	0.6819617	Over	Over	0.9237617	78.362865	3.0080666	Over	26.679456	Over	Over	Over

Over=Over max concentration

The drug sensitivity scores (DSS) of CRCs

Drugs	P1-UC	P1-TC	P2-UC	P3-UC	P3-TC	P4-UC	P4-TC	P5-UC	P6-UC	P6-TC	P7-UC	P8-UC	P9-UC	P10-UC	P11-UC	P12-UC	P13-UC
**Temsirolimus**	Over	Over	Over	Over	Over	Over	Over	Over	Over	Over	Over	Over	Over	Over	Over	Over	0.3977100
**Imatinib**	Over	Over	Over	Over	Over	Over	Over	Over	Over	Over	Over	Over	Over	Over	Over	Over	Over
**Regorafenib**	Over	Over	Over	Over	Over	Over	Over	Over	Over	Over	Over	Over	Over	Over	Over	Over	Over
**Sorafenib Mesylate**	Over	Over	Over	Over	Over	Over	Over	Over	Over	Over	Over	Over	Over	Over	Over	Over	Over
**Palbociclib**	Over	Over	Over	Over	Over	Over	Over	Over	Over	Over	Over	Over	Over	Over	Over	Over	Over
**Ponatinib**	-1.0098458	-0.7253610	0.7248331	0.0331850	-0.0649072	0.9740042	Over	-0.6027533	Over	Over	0.9541245	0.9438237	Over	0.9532617	0.1256989	-0.1591535	-1.9165900
**Vinorelbine**	-1.7611075	Over	Over	Over	-0.1898167	Over	Over	-1.8566629	Over	Over	Over	-0.3518875	Over	Over	Over	Over	-0.7707977
**Cyclophosphamide**	Over	Over	Over	Over	Over	Over	Over	Over	Over	Over	Over	Over	Over	Over	Over	Over	Over
**Ifosfamide**	Over	Over	Over	Over	Over	Over	Over	Over	Over	Over	Over	Over	Over	Over	Over	Over	Over

**Daunorubicin**	0.4187309	0.4919391	0.8494715	0.8706873	0.6419424	1.0876827	0.8964455	0.6362666	1.2833451	1.4200540	0.8416654	0.4285202	Over	0.9698732	0.3401361	0.5585174	-0.3252749
**Idarubicin**	-1.2773139	-1.4167100	-0.1539550	-0.8698250	-1.5926345	1.0993049	-1.1389150	-1.1781990	-0.4584328	-0.4273735	-1.1445080	-1.5739525	-0.9482763	-0.8190097	-1.3951764	-0.8584340	-3.3658040
**Irinotecan**	0.7298441	Over	Over	Over	0.2309863	Over	Over	Over	Over	Over	-0.2277909	-0.0030147	0.1440828	Over	Over	0.0979542	Over
**Anastrozole**	Over	Over	Over	Over	Over	Over	Over	Over	Over	Over	Over	Over	Over	Over	Over	Over	Over
**Tamoxifen**	Over	Over	Over	Over	Over	Over	Over	Over	Over	Over	Over	Over	Over	Over	Over	Over	Over
**Etoposide**	Over	Over	Over	Over	Over	Over	Over	Over	Over	Over	-2.1364174	-1.0784378	Over	Over	Over	Over	Over
**Mercaptopurine**	Over	Over	Over	Over	Over	Over	Over	Over	Over	Over	Over	Over	Over	Over	Over	Over	Over
**Sirolimus**	Over	Over	Over	Over	Over	Over	Over	Over	Over	Over	Over	Over	Over	Over	Over	Over	Over
**Exemestane**	Over	Over	Over	Over	Over	Over	Over	Over	Over	Over	Over	Over	Over	Over	Over	Over	Over
**Mitoxantrone**	-0.4816511	-0.6798907	-0.1640240	-0.2113786	-0.8464167	-0.0922411	-0.3053068	-0.5421133	-0.1213362	0.0148385	1.1192376	Over	0.6938512	-0.1522334	-0.6723158	-0.2617734	Over
**Cladribine**	Over	Over	Over	Over	Over	Over	Over	Over	Over	Over	Over	Over	Over	Over	Over	Over	Over
**Cytarabine**	Over	Over	Over	Over	Over	Over	Over	Over	Over	Over	Over	Over	Over	Over	Over	Over	Over
**Hydroxyurea**	Over	Over	Over	Over	Over	Over	Over	Over	Over	Over	Over	Over	Over	Over	Over	Over	Over
**Gemcitabine Hydrochloride**	Over	0.0395820	Over	Over	0.3116033	Over	Over	-0.2492820	Over	Over	-0.1389632	-0.1607389	Over	Over	Over	Over	Over
**Cisplatin**	0.6705288	Over	0.4547044	1.2007315	0.8193552	Over	Over	0.4695351	Over	Over	0.1653672	Over	1.2006845	Over	Over	1.0574039	Over
**Carboplatin**	Over	Over	Over	Over	Over	Over	Over	Over	Over	Over	Over	Over	Over	Over	Over	Over	Over
**Nedaplatin**	0.2859419	0.0577951	0.1392823	Over	Over	Over	0.1652784	0.7363402	Over	Over	Over	Over	Over	0.1156854	0.5461630	-0.7449608	Over
**Methotrexate**	Over	Over	Over	Over	Over	Over	Over	Over	Over	Over	Over	Over	Over	Over	Over	Over	Over
**Vinblastine sulfate**	Over	Over	Over	Over	-1.3487543	Over	Over	-1.0760439	Over	Over	Over	-0.5527682	Over	Over	Over	Over	Over
**Doxorubicin Hydrochloride**	-0.7143238	-0.5244717	-0.2520288	-0.6242456	-0.9878217	-0.1644799	-0.5079069	-0.7893006	0.7051954	0.9212353	-0.6134421	-1.0983688	-0.4395729	-0.2865213	-0.5086882	-0.6834771	Over
**Epirubicin Hydrochloride**	-0.5729531	-0.0715592	0.0027285	0.3263125	-3.3767971	0.2783642	0.0611210	-0.0361793	1.2403989	1.1566031	0.5569213	Over	1.1351212	0.2358002	0.2166290	0.3329854	Over
**Pemetrexed**	Over	Over	Over	Over	Over	Over	Over	Over	Over	Over	Over	Over	Over	Over	Over	Over	Over
**Pirarubicin**	0.8079603	Over	1.1680255	1.1832347	0.3309962	0.2261034	0.1437126	0.5660898	Over	Over	Over	Over	Over	0.1543220	Over	0.2193129	Over
**Hydeoxycamptothecin**	0.1770327	0.4923644	Over	1.2163915	0.0035719	Over	2.0255330	-0.6389664	Over	Over	Over	Over	Over	Over	0.7415610	1.1811021	Over
**Mitomycin**	2.2250524	3.2173925	Over	Over	2.4623682	Over	Over	-0.0371992	2.0891221	2.1775818	2.4315063	Over	Over	2.2449302	Over	2.4850206	Over
**Sunitinib**	-0.9196423	-0.8877153	-0.999930	Over	-0.9658326	0.3100338	0.0201715	-0.2654086	Over	Over	-0.9269429	Over	Over	Over	Over	Over	Over
**Everolimus**	Over	Over	Over	Over	Over	Over	Over	Over	Over	Over	Over	Over	Over	Over	Over	Over	Over
**Paclitaxel**	Over	Over	Over	Over	Over	Over	Over	-1.7838059	Over	Over	0.6267131	-2.5193792	Over	Over	Over	Over	Over
**Docetaxel**	Over	Over	Over	Over	Over	Over	Over	-1.7432223	Over	Over	Over	-2.7631939	Over	Over	Over	Over	Over

**Olaparib**	Over	Over	Over	Over	Over	Over	Over	Over	Over	Over	Over	Over	Over	Over	Over	Over	Over
**Erlotinib**	Over	Over	Over	2.1649941	Over	Over	Over	Over	Over	Over	Over	Over	Over	Over	Over	Over	Over
**Afatinib**	Over	Over	5.8940258	Over	2.7564343	Over	2.8348050	2.9393244	Over	Over	Over	Over	0.0231624	Over	Over	Over	Over
**Crizotinib**	1.0058638	Over	0.3945723	0.5136836	0.1507481	0.3547856	0.2899167	0.3848220	Over	0.4531775	0.3381270	Over	Over	0.4157482	Over	0.3689511	Over
**Vemurafenib**	Over	Over	Over	Over	Over	Over	Over	Over	Over	Over	Over	Over	Over	Over	Over	Over	Over
**Trametinib**	Over	Over	Over	Over	Over	Over	Over	Over	Over	Over	Over	Over	Over	Over	Over	Over	Over
**Belinostat**	-0.0287275	-0.2684986	0.2737241	1.1259001	0.0668985	0.9906817	0.6270259	0.4124235	0.8574485	0.7131350	0.3985724	0.2910726	0.6205277	1.0959682	0.1548431	0.8063011	Over
**Bleomycin**	Over	Over	Over	1.6408427	Over	Over	Over	Over	Over	Over	Over	Over	1.4545942	Over	Over	Over	Over
**Capecitabine**	-0.1777567	Over	Over	1.1884067	Over	Over	Over	0.2065992	Over	Over	Over	0.1630459	Over	Over	Over	Over	Over
**Doxifluridine**	Over	Over	Over	Over	Over	Over	Over	Over	Over	Over	Over	Over	Over	Over	Over	Over	Over
**Oxaliplatin**	Over	Over	Over	Over	Over	Over	Over	Over	Over	Over	Over	Over	Over	Over	Over	Over	Over
**Floxuridine**	Over	Over	Over	Over	Over	Over	Over	Over	Over	Over	Over	Over	Over	Over	Over	Over	Over
**Fludarabine**	-0.4639038	-0.7357555	Over	0.8544110	Over	Over	Over	Over	-0.2702802	-0.1783048	Over	Over	Over	Over	Over	Over	Over
**Dacarbazine**	Over	Over	Over	0.5868642	Over	Over	Over	Over	Over	Over	Over	Over	Over	Over	Over	Over	Over
**Topotecan**	Over	Over	-1.6567069	-0.4615745	-0.7227286	-1.9192037	Over	Over	Over	-0.8174096	-2.4683098	-1.9702902	Over	Over	Over	-0.2265958	Over
**Axitinib**	Over	Over	Over	Over	0.0581144	Over	Over	Over	Over	Over	Over	Over	Over	Over	Over	Over	Over
**Bortezomib**	-1.3059180	-1.1842857	-1.8620821	-0.9881760	-0.7817087	-0.5344957	-0.7959706	-0.9546979	-0.9031799	-0.8369635	-0.7544669	-0.6408820	-1.4530873	-1.0494981	-0.9158309	-1.0092502	-0.9200738
**Dasatinib**	0.7062609	0.6408851	1.1824540	Over	0.3657684	Over	Over	0.0578342	Over	Over	0.6731422	-0.3254171	Over	Over	0.3814397	Over	Over
**Gefitinib**	Over	Over	Over	Over	Over	Over	Over	Over	Over	Over	Over	Over	Over	Over	Over	Over	Over
**Ibrutinib**	Over	Over	Over	Over	Over	Over	Over	Over	Over	Over	Over	Over	Over	Over	Over	Over	Over
**Lapatinib**	Over	Over	2.3211114	Over	2.2207212	Over	Over	Over	Over	Over	1.9553485	1.5185487	-3.3067803	Over	Over	Over	Over
**Nilotinib**	Over	Over	Over	Over	Over	Over	Over	Over	Over	Over	Over	Over	Over	Over	Over	Over	Over
**Pazopanib**	Over	Over	Over	Over	Over	Over	Over	Over	Over	Over	Over	Over	Over	Over	Over	Over	Over
**Trametinib**	Over	Over	Over	Over	Over	Over	Over	Over	Over	Over	Over	Over	Over	Over	Over	Over	Over
**Dabrafenib**	Over	Over	Over	Over	Over	Over	Over	Over	Over	Over	1.0864594	Over	Over	Over	Over	Over	Over
**Vorinostat**	Over	Over	Over	Over	Over	Over	Over	Over	Over	Over	Over	Over	Over	Over	Over	Over	Over

Over=Over max concentration
